# From immune checkpoint blockade to metabolic ecosystem intervention: A new perspective on ammonia‐mediated remodelling of tumour immunity

**DOI:** 10.1002/ctm2.70792

**Published:** 2026-09-16

**Authors:** Yu Li, Yun Zhu, Jian Gu

**Affiliations:** ^1^ Collaborative Innovation Centre for Cancer Personalised Medicine and Hepatobiliary Centre The First Hospital Affiliated with Nanjing Medical University Nanjing China; ^2^ Department of General Surgery The Affiliated BenQ Hospital of Nanjing Medical University Nanjing China

In recent years, immune checkpoint blockade (ICB) therapy has profoundly reshaped the landscape of cancer treatment and has provided durable clinical benefits for a subset of patients. However, accumulating evidence indicates that the efficacy of immunotherapy is not solely determined by the ability of immune cells to be effectively activated, but is also profoundly influenced by the dynamic evolution of the tumour metabolic microenvironment. Current immunotherapeutic strategies primarily focus on classical immune checkpoints, such as programmed death‐1/programmed death‐ligand 1 (PD‐1/PD‐L1) and cytotoxic T‑lymphocyte‐associated protein 4 (CTLA‐4), whereas the metabolic reprogramming induced during treatment and its feedback regulation of the immune ecosystem remain incompletely understood.^1^ Based on our recent findings, we propose that ammonia should not be regarded merely as a metabolic waste product, but rather as a critical metabolic mediator linking tumour metabolic dysregulation, immune cell adaptation and therapeutic resistance. Furthermore, we discuss its potential clinical implications in treatment response evaluation and the development of rational combination therapies.

## 1 METABOLIC CHALLENGES IN THE ERA OF IMMUNOTHERAPY: FROM IMMUNE ACTIVATION TO METABOLIC ECOSYSTEM REMODELLING

The tumour microenvironment (TME) is not merely a passive context in which immune responses occur, but rather a dynamic ecosystem composed of tumour cells, immune cells, stromal components and diverse metabolites.^2^ Through metabolic reprogramming of glucose, glutamine, lipids and other amino acid pathways, tumour cells not only fulfil their biosynthetic and energetic demands but also continuously reshape local nutrient availability and metabolite landscapes.^3^ This metabolic competition and stress within the TME can impair the proliferation and effector functions of antitumor T cells.^4^ while simultaneously conferring selective adaptive advantages to immunosuppressive populations, including regulatory T cells (Tregs) and myeloid‐derived suppressor cells (MDSCs).^5^

Current clinical immunotherapy strategies predominantly focus on classical immune checkpoints, including PD‐1/PD‐L1 and CTLA‐4, with the primary goal of releasing inhibitory signals and restoring antitumor immune activity. However, immunotherapy itself can profoundly reshape the metabolic ecosystem within the TME. Treatment‐induced tumour cell death, proteolytic degradation, metabolite release and the re‐establishment of metabolic competition among immune cell populations may collectively remodel the post‐treatment immune landscape and contribute to the development of acquired resistance.^6^ Therefore, future advances in cancer immunotherapy will require not only the identification of additional immune targets, but also precise modulation of the dynamically evolving metabolic ecosystem during treatment. Integrating metabolic interventions with ICB may provide a synergistic strategy to enhance therapeutic durability and overcome resistance.

## 2 RECONSIDERING AMMONIA: FROM A METABOLIC WASTE PRODUCT TO A REGULATOR OF THE TUMOUR IMMUNE ECOSYSTEM

Among the diverse factors involved in remodelling the tumour metabolic microenvironment, ammonia has historically received limited attention. Ammonia is primarily generated through glutamine catabolism, amino acid deamination and protein degradation, and has traditionally been regarded as a toxic metabolic byproduct that requires rapid detoxification through the urea cycle.^7^ Consequently, previous studies have largely focused on its pathological roles in liver dysfunction, hyperammonaemia and neurotoxicity, whereas its potential implications in tumour immune regulation and therapeutic responses remain poorly understood.^8^ Unlike well‐characterised immunoregulatory metabolites, such as lactate, adenosine and tryptophan‐derived metabolites, whether ammonia can actively shape immune cell composition, alter intercellular metabolic competition and contribute to immunotherapy resistance remains to be systematically elucidated.

**FIGURE 1:**
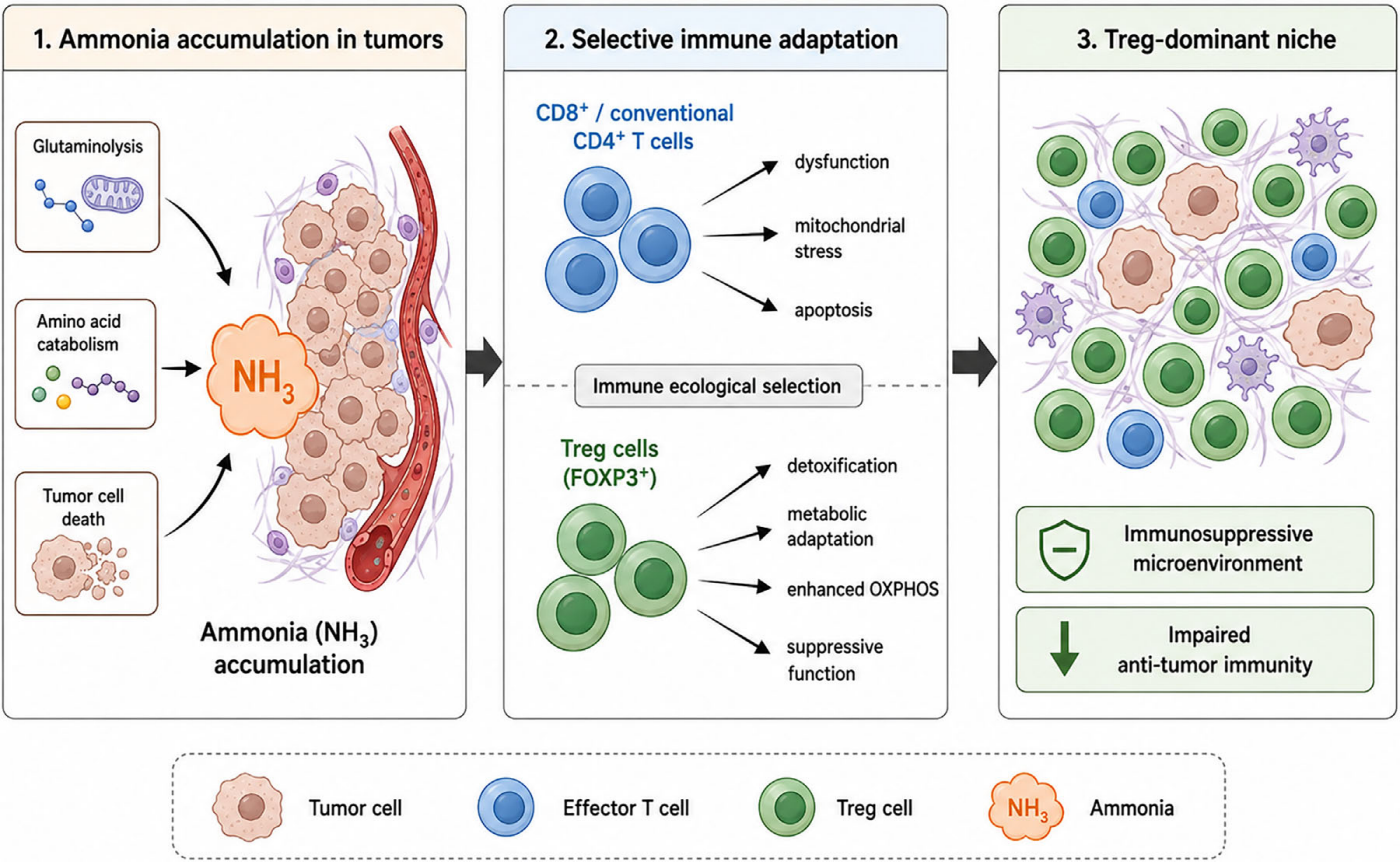
Ammonia reshapes the tumour immune ecosystem.

Our findings suggest that tumour‐derived ammonia is not merely a passive indicator of metabolic abnormalities, but rather an active mediator that reshapes the tumour immune ecosystem. Elevated ammonia exposure does not exert uniform effects across immune populations; instead, it differentially influences metabolic adaptation capacity and competitive fitness among distinct immune cell subsets. Effector CD4^+^ and CD8^+^ T cells are more vulnerable to ammonia‐induced metabolic stress, whereas Tregs maintain survival through ammonia detoxification and metabolic reprogramming, thereby further enhancing their immunosuppressive capacity.^9^ Therefore, the impact of ammonia is not simply a global suppression of immune activity, but rather a selective remodelling process that favours immune cell populations with superior adaptive capacity, ultimately driving the tumour immune ecosystem toward Treg dominance. Collectively, ammonia may represent a critical metabolic mediator linking tumour metabolic dysregulation with immune functional remodelling, providing a new ecological framework for understanding immunotherapy resistance (Figure 1).

## 3 THERAPY‐INDUCED AMMONIA METABOLIC REMODELLING: LINKING INITIAL RESPONSE TO ACQUIRED RESISTANCE

Anti‐PD‐1 therapy restores effector T‐cell function, thereby promoting tumour cell killing, antigen release and immune activation. However, tumour cell death induced by effective immune responses may simultaneously increase local ammonia availability through enhanced deamination processes. The resulting elevation of intratumoral ammonia levels may, in turn, support the survival and functional maintenance of Tregs. These observations suggest that robust initial therapeutic responses and subsequent acquired resistance may not represent independent events, but rather distinct phases of the same dynamic evolutionary process. In other words, while immunotherapy activates antitumor immunity, it may also progressively establish metabolic conditions that favour the reconstruction of an immunosuppressive TME.

This mechanism provides a potential explanation for why “immune‐inflamed” (hot) tumours can still develop acquired resistance despite their favourable initial responsiveness. Hot tumours are typically characterised by increased T‐cell infiltration and heightened immune activity, which contributes to improved early responses to immunotherapy.^10^ However, we propose that stronger initial immune‐mediated tumour killing may also result in more extensive tumour cell death and ammonia release, subsequently promoting Treg accumulation and re‐establishment of an immunosuppressive microenvironment. Within this framework, enhanced early therapeutic responses may paradoxically create metabolic conditions that facilitate subsequent resistance, representing a form of “response‐associated metabolic resistance.” Thus, early response and later resistance are not necessarily contradictory outcomes, but may instead be continuously driven by the same therapy‐induced metabolic feedback loop.

**FIGURE 2:**
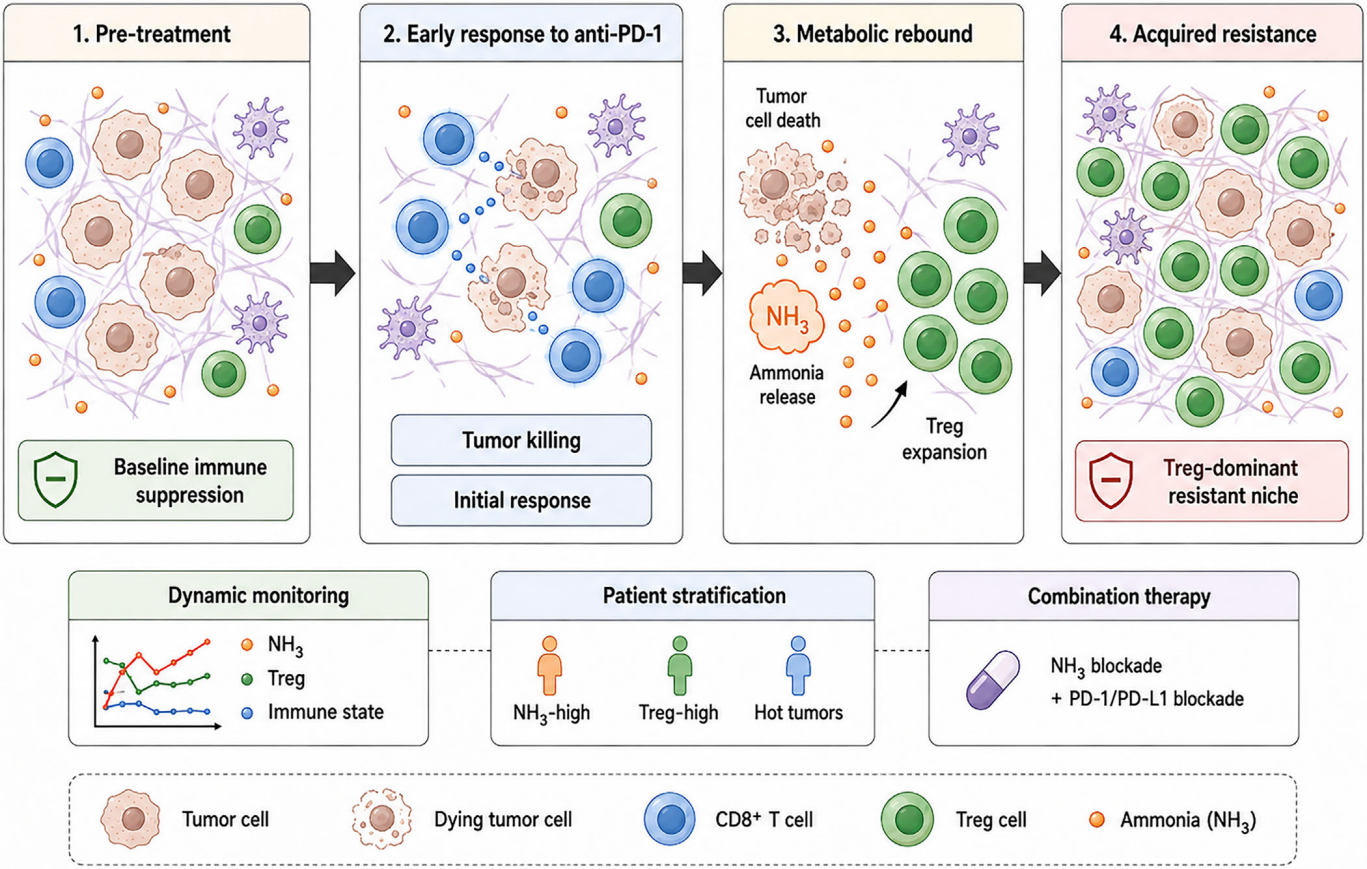
Therapy‐induced ammonia remodelling links initial response to acquired resistance.

Collectively, these findings highlight that immunotherapy not only relieves classical immune inhibitory pathways but also continuously remodels the tumour metabolic ecosystem. For patients who exhibit substantial initial responses followed by disease progression, post‐treatment ammonia accumulation and Treg re‐expansion may represent critical events underlying acquired resistance. Therefore, evaluation of immunotherapy efficacy should extend beyond tumour regression and effector T‐cell activation, and should also consider whether a new immunosuppressive metabolic niche emerges during treatment adaptation (Figure 2).

## 4 FROM DYNAMIC METABOLIC MONITORING TO PRECISION ECOSYSTEM INTERVENTION: A NEW DIRECTION FOR CANCER IMMUNOTHERAPY

The tumour metabolic microenvironment is not a static entity, but rather continuously evolves in response to disease progression and therapeutic pressure. Therefore, future metabolic therapies should not be limited to identifying single, fixed metabolic targets, but instead should be dynamically tailored according to tumour stage, immune status and metabolic characteristics. During the immunotherapy‐sensitive phase, metabolic interventions may aim to enhance effector T‐cell function and sustain antitumor immunity. In contrast, during the emergence of therapeutic resistance, precise modulation of ammonia, polyamine metabolism, and other immunosuppressive metabolic pathways may provide new opportunities for overcoming resistance. The identification of the ammonia–Treg axis further suggests that metabolites are not merely byproducts accompanying tumour growth, but can function as active regulators that determine immune cell competition and therapeutic outcomes.

Future efforts should focus on establishing integrated monitoring platforms capable of capturing dynamic changes in the tumour metabolic ecosystem. By incorporating spatial metabolomics, metabolic imaging, liquid biopsy approaches and artificial intelligence‐assisted multimodal analyses, it may become possible to longitudinally evaluate tumour metabolic states, immunotherapy responses and resistance risks. Building upon such dynamic metabolic profiling, personalised combination strategies could be developed according to individual metabolic vulnerabilities, including the integration of ammonia production inhibition, enhanced ammonia clearance or blockade of related metabolic pathways with immune checkpoint inhibitors. Moving beyond static biomarkers toward dynamic metabolic monitoring‐guided therapeutic decision‐making may represent a critical step toward precision immunotherapy.

Collectively, ammonia may represent a long‐overlooked metabolic regulatory dimension in cancer immunotherapy. Beyond reflecting tumour metabolic dysregulation, ammonia may actively reshape competitive interactions among immune cell populations, thereby linking initial therapeutic responses with subsequent resistance evolution. In the future, integration of dynamic metabolic monitoring, spatial profiling and artificial intelligence‐assisted predictive modelling may enable more precise characterisation of the evolving tumour immune ecosystem and facilitate rational combinations of immunotherapy and metabolic interventions. Reframing immunotherapy resistance as a dynamic process jointly driven by immune signalling and metabolic ecosystem remodelling may provide a new conceptual framework for patient stratification, response monitoring and optimisation of combination therapeutic strategies.

## CONFLICT OF INTEREST STATEMENT

The authors declare no conflict of interest.

## FUNDING INFORMATION

This article received no specific funding.

## ETHICS STATEMENT

Ethics approval was not required for this article, as it discusses previously published research and reports no new studies involving human participants or animals.

## Data Availability

Data sharing is not applicable to this article, as no datasets were generated or analysed during the current study.
